# Impact of increasing market pig weights in North America: a comprehensive review

**DOI:** 10.1186/s40104-026-01453-4

**Published:** 2026-06-27

**Authors:** Justice Bless Dorleku, Isaac Hyeladi Malgwi, Tawanda Tayengwa, Benjamin Mark Bohrer, Marcio de Souza Duarte, Ángela Cánovas, Luigi Faucitano, Manuel Juárez

**Affiliations:** 1 Lacombe Research and Development Centre, Agriculture and Agri-Food Canada, Lacombe, AB T4L 1W1 Canada; 2 Department of Animal Biosciences, University of Guelph, Guelph, ON N1G 2W1 Canada; 3 Department of Agronomy, Food, Natural Resources, Animals and Environment, University of Padova, Viale Dell’Università 16, Legnaro, Padua, 35020 Italy; 4 Department of Animal Sciences, The Ohio State University, Columbus, OH 43210 USA; 5 Department of Animal Science, Federal University of Viçosa, Viçosa, MG 36570-900 Brazil; 6 Centre for Genetic Improvement of Livestock, Department of Animal Biosciences, University of Guelph, Guelph, ON N1G 2W1 Canada; 7 Sherbrooke Research and Development Centre, Agriculture and Agri-Food Canada, Sherbrooke, QC J1M 0C8 Canada

**Keywords:** Animal welfare, Carcass traits, Genomic selection, Heavy pig, Heritability, Immunocastration, Meat quality, Swine performance

## Abstract

The North American swine industry is shifting towards raising pigs to heavier weights, partly due to the potential for higher profitability for pork processors. However, producers may incur losses due to feed and transportation costs associated with heavy-weight pigs. In some cases, these animals can fall outside grid-based grading specifications, resulting in discounts for carcasses that are too heavy or too lean/too fat. Furthermore, increasing weight raises concerns regarding nutritional requirements, feed efficiency, environmental sustainability, welfare issues, carcass composition, chilling rate, meat quality, and implications for genomic selection. Variation exists for growth performance traits and carcass composition across genotypes. In this review, we summarise the biological, physiological, and genetic mechanisms underlying the effect of increasing weight in contemporary pig production. A comprehensive understanding of animal performance, animal welfare, carcass traits and genetic regulations in heavy-weight pigs will be significant for refining breeding goals, precision feeding strategies and handling practices.

## Introduction

**Fig. 1:**
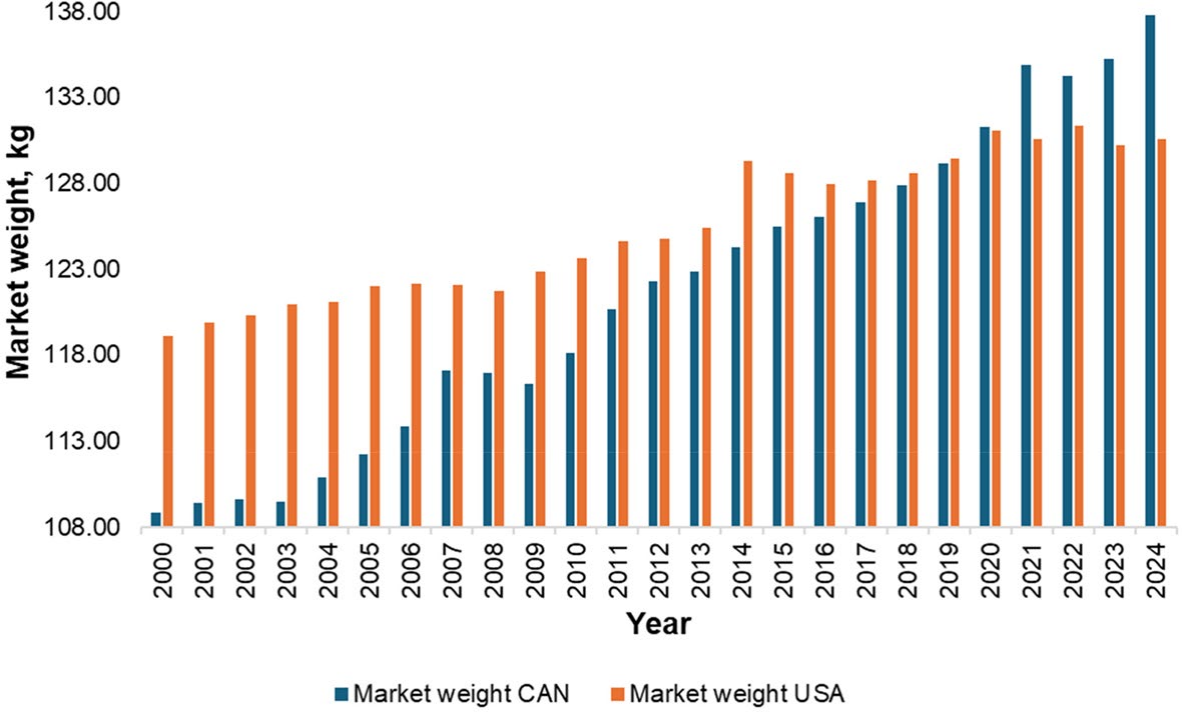
The trend of average pig market weight over two decades (2000 to 2024) in North America. Market weight for the USA was directly derived from USDA ERS [2]. For Canada, hot carcass weight (HCW) was derived from AAFC [1] and converted to market weight using a conversion factor of 80% to convert HCW to market weight

Nearly a decade ago, pigs weighing over 130 kg were often classified as heavy [1, 2]. However, recent industry trends on market weight (i.e., live ending weights at the time of slaughter) suggest the definition of “heavy” should be re-evaluated. Over the past two decades, the North American pork industry has experienced notable increases in average market pig weights (Fig. 1) [3, 4]. Such a shift in production strategy affects the global pork market, as the USA and Canada are among the top pork producers worldwide and heavily involved with the international trade of pork [5]. Between these two countries, the average market weight rose from 114 kg in 2000 to 134 kg in 2024. Canada had the largest increase, with an increase in average market weight of 26.5% (28.9 kg) [3] from 2000 to 2024, reflecting changes in production and shifts in market demand [6]. In contrast, the USA exhibited smaller changes in average market weight compared with Canada, with the average market weight increasing by 11.5 kg, representing a 9.7% gain over the same period [4]. The observed changes in average market weight directly correspond with trends for heavier hot carcass weights (HCW) by roughly 0.4 kg/year (Fig. 1) [7–12]. The more rapid increase in market hog weights observed in Canada relative to the USA appears to be driven primarily by structural and economic factors, including Canada’s export-oriented production system, integration with the U.S. finishing and slaughter capacity, and marketing strategies that favour heavier feeder and market hogs, rather than by fundamental differences in biological growth potential alone.

Table 1 provides projections for market weights to 2050, based on historical data from 2000 to 2024 [3, 4], with Canada expected to record the greatest annual gains (1.2 kg), reaching 169 kg by 2050, if the trend continues. Growth patterns are influenced by marketing strategies, industrialisation, and regional economic conditions, with local practices and global demands shaping production outcomes. Marketing strategies can minimise weight variation and improve growth performance [1, 13, 14].

**Table 1:** Projected market weights for pigs from North America^1^

Country	Annual increase, kg	2030	2040	2050
USA^2^	0.5	133.47	138.27	143.07
Canada^2^	1.2	144.97	157.00	169.03

^1^Projections were created based on the average market weight reported per year from 2000 to 2024. Measured in kg, and a conversion factor of 80% was used to convert hot carcass weight (HCW) to market weight for Canada

^2^For Canada, HCW was adapted from AAFC [3] and converted to market weight. Market weight for the USA was directly derived from USDA ERS [4]

The increase in market pig weights can be attributed to the economic benefits of diluting fixed production costs over heavier pigs and advancements in genetic selection for leaner and faster-growing pigs [2]. Fixed costs associated with housing, labour, and transportation can be distributed over greater carcass weight, improving overall economic efficiency on a per-unit basis. Furthermore, packing plants in North America are highly consolidated and optimised for high-throughput processing; heavier pigs increase carcass yield per animal, maximising plant efficiency and profitability. Pricing systems in North America often incentivise lean yield and carcass weight, encouraging producers to market pigs at heavier endpoints. From a supply chain perspective, producing heavier pigs reduces the number of animals required to meet total pork demand, which can lower logistical pressures. Additional economic benefits may arise from improved immune resilience [2], as animals experiencing lower stress or enhanced immune function may require fewer health interventions. This reduction in disease susceptibility can lower certain vaccine-related costs and improve the overall efficiency of health expenditures, ultimately contributing to greater profits when pig prices are high or when finishing feed prices are relatively low [15].

The trend towards the production of heavier pigs raises critical questions about animal welfare and environmental impact. Reaching heavier weights may create challenges for pig processing facilities, namely, handling and transportation systems. Furthermore, welfare assessments for heavier pigs often lack standardised and validated methodologies, making it difficult to quantify their behavioural needs or emotional states [16]. From an environmental impact perspective, it has been demonstrated that heavy-weight pigs increase the environmental impact of pig production due to less favourable feed efficiency [17]; however, this can vary based on the production system employed, and therefore, additional information is needed to understand specific systems.

Currently, breeding programmes target market pig weight to maximise genetic gains at that stage of animal development. Given that the ‘breeding programme’ refers to the genetic evaluation conducted by commercial genetics companies at a specified target weight, the impact of marketing pigs at heavier weights is unknown, as is the potential need to adjust existing selection indices. Innovations in genetic selection, such as advanced genomic technologies, are now being employed to enhance carcass traits, including lean yield and meat palatability. This review examines the drivers, implications, challenges, and future research needs related to increasing pig body weight at heavier market weights. Specifically, it discusses the global trend, with a focus on North America, the economic impact, and nutrition, as well as the effects of increased weight on growth performance, environmental impact, animal welfare, carcass composition, pork quality traits, and potential implications for genetic selection.

### Economic impact of increasing market pig weights

Although heavier HCW can improve carcass yield and offer processing advantages for packers, the economic return of rearing heavier pigs is not guaranteed for the production sector and can often be negative. An economic analysis comparing pigs slaughtered at market weights of 120 and 140 kg showed that the net margin per pig space often decreased due to longer rearing periods, reduced turnover for barns, and greater feed costs. As a result, the number of kg sold per pig space declined, reducing overall profitability for the producer. This study concluded that increasing market pig weights can only provide greater profits when pig prices are high or when finishing feed prices are relatively low [15].

This disconnect highlights that the added value of heavier pigs often benefits the packer more than the producer, particularly in systems where grading grids and pricing structures do not adequately compensate for increased input costs. In contrast, vertically integrated systems, where the same company controls both production and processing sectors, are better positioned to capture the benefits of heavier carcasses. For independent producers, unless there are changes to carcass grading limits or pricing mechanisms, the push for heavier pigs may not translate into increased profitability and could, in fact, result in financial losses.

A review by Kim et al. [6] concluded that increasing market pig weight enhances net profits for producers despite higher expenses. Similarly, optimisation models developed in Denmark revealed that raising market pig weights by 5 kg increases gross income by US$217,000 but decreases profit per kg by US$0.037/kg. However, further increases of 10 and 15 kg reduce per kg prices by US$0.076/kg and US$0.109/kg, respectively [18]. These studies suggest that total profit can increase with heavier pigs, but profitability per kg declines, and production costs rise. Overall, in the absence of market structures or pricing grids that adequately compensate for heavier carcasses, the financial advantages of increased weight may bypass the production sector, instead favouring packers or integrated operations.

From a supply and consumption standpoint, the United States produces approximately 28.5 billion pounds of pork annually, with continued growth in both domestic use and exports [4]. Pork remains a major protein source, although per capita consumption in North America has been relatively stable rather than rapidly increasing, meaning that production gains are often absorbed through export markets or value-added processing. In contrast, Canada is highly export-oriented, with approximately 70% of pork production destined for international markets [19]. This export dependence incentivises efficiency improvements, including heavier market weights, to remain competitive globally. Increasing carcass weights enhances total output without proportionally increasing fixed costs, thereby improving production efficiency and total industry value. However, these gains are not evenly distributed across the supply chain. At the processing level, large, highly consolidated slaughter companies benefit significantly from heavier pigs because greater carcass weights increase throughput efficiency and product yield per animal. Consumer preferences, however, introduce complexity. While heavier carcasses improve processing efficiency, they may produce larger primal cuts that are less aligned with retail portion sizes or foodservice specifications. In North America, where consumption growth is modest, the industry has increasingly relied on further processing and product diversification to maintain demand rather than simply increasing fresh pork consumption. This creates a balancing act between biological efficiency (heavier pigs) and market acceptability (portion size, fat composition, and product formats).

### Effects of increasing slaughter weight on growth performance and feed efficiency

As pigs are reared to heavier weights, their growth performance is influenced by various factors, including genetics, diet, space allowance, and production strategies. These factors collectively affect average daily gain (ADG), average daily feed intake (ADFI), and gain-to-feed ratio (G:F), which are critical production metrics for economic feasibility and resource utilisation. Several studies have explored the impact of pigs’ body weight on growth performance; however, this review section focused exclusively on research conducted on market pig weights ranging from 100 to 215 kg and belonging to both lean-type and fat-type pigs, such as those reared for specialty ham production. A reason for these divergent production focuses is the differences in physiological maturity at similar weights, where the concept of early- and late-maturing breeds/genotypes comes into play. Besides the lean-type and fat-type categories, there is also variation in the maturity rate within given production focuses.

It has been reported that in lean-genotype pigs, growth performance parameters, such as ADG and G:F, subtly change with an increase in weight between 110 and 140 kg. Whereas, ADG and G:F experience markedly erosion in fat-type pigs as market pig weight increased within this weight range [20]. This indicates that the effect of increasing weights on growth performance is genotype-dependent.

Wensley et al. [21] found improved ADG in early-maturing pigs until 50 kg (94 d of age). In contrast, in late-maturing pigs, improved ADG started from around 100 kg (136 d of age) until 140 kg (170 d of age), with maximum ADG occurring at ~ 88 kg in early-maturing and 119 kg in late-maturing pigs. The authors also found that the cumulative ADFI from 76 to 170 d of age was higher for early-maturing pigs than for late-maturing ones, resulting in an improved G:F for late-maturing pigs.

As pigs mature and reach heavier weights, their G:F generally declines, with growing pigs achieving a G:F better than 0.50, whereas finishing pigs often drop below 0.33 [22]. This view is supported by an exhaustive review paper reporting a greater feed intake, but also slower growth rates and reduced feed efficiency in heavier market pigs compared to lighter ones [14]. When pigs are reared to heavier weights, their growth patterns tend to change, often in terms of decreasing ADG [14] and increasing ADFI [23–26]. This change is primarily driven by the physiological shifts and management practices associated with aiming for heavier market weights. Stender [22] reported that as pigs grow heavier, their feed efficiency declines due to increased maintenance energy requirements and a shift in gain composition towards less efficient fat production, leading to a decrease in ADG. Malgwi et al. [27] reported that rearing pigs to 160 ± 16 kg weight at a younger age resulted in a 32% increase in ADG compared to the control group. On the other hand, the study of the effects of slaughter age (from 160 to 175 d) within a 122 to 136 kg weight range showed a 4.5% increase in ADFI over the 15-d interval [28]. Similarly, based on their review of findings from a number of studies, Wu et al. [2] concluded that for every 10 kg increase in weight, ADFI can increase by an average of 78.1 g.

The above findings generally suggest that pigs become less efficient and grow more slowly at heavier weights, which is an expected pattern as muscle deposition plateaus and fat accumulation, which requires more energy, becomes predominant. However, past studies on ADG in pigs weighing between 100 and 215 kg have produced conflicting results, ranging from increased to decreased ADG as the pigs’ weight increased [25, 26, 28–33]. Specifically, Latorre et al. [29] found that increasing weight from 116 to 133 kg negatively impacted ADG, with a decrease of 38 g/d for every 10 kg increase in weight. A similar discrepancy was also highlighted by Wu et al. [2], who reported improved ADG in half of the reviewed studies and an ADG decline in the other half. These differences underscore the impact of genotype, management, and environmental factors on growth performances at heavier weights, highlighting the need for standardised experimental designs.

### Nutritional strategies for heavy-weight finishing pigs

Increasing market weights, driven by genetic improvements in lean growth and feed efficiency, have shifted nutrient requirements during late finishing [34, 35], yet dose–response data for pigs above 135 kg remain limited. In recent years, extensive research has focused on the impact of reductions in dietary net energy on growth performance and carcass characteristics of market pigs, mainly due to the costs of energy-dense ingredients [36, 37]. Smit et al. [36] indicated that reducing dietary net energy level from 2.4 to 2.1 Mcal/kg increased ADFI by ~ 43 g per 0.1 Mcal net energy per kg reduction. Similarly, reducing net energy levels based on the National Research Council's ingredient net energy values [38] resulted in a significant linear increase in ADFI of ~ 0.023 kg/d (23 g/d) [37]. Interestingly, reductions in dietary energy density did not influence ADG in pigs slaughtered at 124 kg [36], while a decline in net energy levels decreased ADG in 137 kg pigs [37]. The discrepancy in ADG, despite similar increases in ADFI, may be a result of differences in market pig weights, energy partitioning, and metabolic efficiency, with heavier pigs being less able to convert additional feed into lean growth. While Smit et al. [36] reported no effect of reduced dietary net energy on lean meat yield and backfat thickness (BFT), Royall et al. [37] observed increased lean meat yield and decreased BFT as net energy was reduced.

Optimising dietary amino acid balance is central to efficient growth, carcass composition, and economic performance in heavy-weight finishing pigs. Standardised ileal digestible (SID) lysine and other essential amino acids (methionine, threonine, tryptophan) should be formulated using the ideal protein concept, with ratios maintained relative to lysine and adjusted across multiple diets for different weight ranges to match changing requirements [34, 35, 39]. As pigs grow, appetite increases while protein requirements as a proportion of the diet decline, highlighting the importance of phase-feeding to avoid oversupply, excess nitrogen excretion, and feed cost [39]. Currently, SID lysine remains a reliable nutritional intermediate, though requirements vary with body weight, sex, genotype, and ambient conditions [40–42]. Meta-analyses results recommend 0.70% and 0.75% SID lysine for barrows and gilts, respectively, at 100 to 135 kg weight [43]. In addition, the effects on the ADG are variable due to genotypic differences in amino acid utilisation, particularly in lysine efficiency and protein deposition [44]. Furthermore, when dietary SID lysine is supplied in excess of the animal’s requirement, the surplus cannot be utilised for protein accretion and must be deaminated, which increases nitrogen excretion [39]. Under such conditions, feed is used less efficiently, resulting in a higher feed intake relative to body weight gain (i.e., poorer feed conversion ratio). As such, slight adjustments in dietary energy density influence feed intake and growth through reductions in net energy, leading to compensatory intake (43 g per 0.1 Mcal/kg or 23 g/d per 2% reduction) [36, 37], improved ADG at 124 kg but declining near 137 kg as energy partitioning shifts toward maintenance and fat deposition. In addition, protein-sparing strategies, such as reducing crude protein with crystalline amino acids, maintain ADG, ADFI, and feed efficiency up to 122 kg [45], but their efficiencies above 130 kg are limited by reduced nitrogen retention [26].

Micronutrient and lipid manipulations, including vitamin E (up to 200 ppm all-rac-α-tocopheryl acetate) and distillers corn oil, yield modest and inconsistent effects on performance and carcass composition [46, 47]. Feeding management strategies, including ad libitum and restricted regimes, have been applied to heavy-weight pigs intended for Protected Designation of Origin dry-cured ham production (differences among restricted/realimentation feeding regimes exist among dry-cured ham production systems) and demonstrated a significant impact on growth performance [25–27]. However, Protected Designation of Origin pig production differs markedly from North American market pigs as it employs distinct nutritional strategies and involves pigs with different genetic backgrounds. Restricted feeding is generally not recommended for market-weight pigs unless combined with electronic feeders and precision feeding technologies. Ad libitum feeding, while convenient, may increase feed waste [14], whereas restricted feeding can help control excessive fat deposition. Nevertheless, modern feeding equipment mitigates this issue, and feed waste is routinely (an estimated 5% allowance) accounted for in diet formulation according to NRC [38]. Consequently, phased, precision-feeding programs tailored to heavy-weight endpoints can improve efficiency while supporting sustainability goals.

### Environmental and sustainability implications of heavy-weight pigs

The production of heavy-weight pigs has a significant environmental impact, largely due to less favourable feed efficiency associated emissions. In this regard, some authors have indicated that greenhouse gas emissions, such as methane and nitrous oxide from manure and enteric fermentation during the growing-finishing phase, are primarily due to high levels of feed consumption and inefficient feed utilisation [48, 49]. According to del Rosario Villavicencio-Gutiérrez et al. [17], when compared with 116 kg pigs, the production of 155 kg pigs increases the environmental impact by 60% to 75% (on a per kg basis using a life cycle analysis approach). Generally, feed production, including crop cultivation and manufactured feed, is the dominant contributor to environmental impacts in heavy-weight pig production, accounting for over 62.5% of global warming [50] and more than 74% across six key impact categories, such as agricultural land occupation, climate change, freshwater ecotoxicity, human toxicity, marine ecotoxicity, and water depletion [17], making it the primary critical point across the entire feed chain [51]. In production systems with stable domestic consumption and limited exports, increasing market pig weights reduces the total number of animals required, which may lower certain environmental burdens (e.g., facility use, manure volume, and transport emissions), but the net impact on sustainability depends on changes in resource use efficiency, days on feed, and emissions intensity per unit of pork produced.

Given that feed production is the primary critical point for greenhouse gas in pig production, it is essential to assess the contribution of feed efficiency and the impact of feed composition on heavy-weight pigs. Notably, some researchers have reported that, regardless of sex, as pigs reach heavier weights, the carbon footprint associated with feed intake, measured per kg of carcass growth and carcass weight, increases significantly during the finishing phase [52]. The authors projected an increase in the carbon footprint of feed intake during the finishing phase, estimating a rise of 5.9 g CO_2_-eq/kg carcass growth for pigs with weights ranging from 110 to 148 kg housed individually and fed ad libitum a nutrient-rich diet. In contrast, pigs housed in groups and fed ad libitum a commercial diet with a weight range of 99 to 138 kg showed a lower increase of 4.4 g CO_2_-eq/kg carcass growth. This suggests that the higher carbon footprint of heavier pigs may be partly attributed to nutrient-rich diets, highlighting the need for different ingredients and different energy levels in diets, or genetic selection for animals with improved feed efficiency and lower requirements. Understanding these interactions would effectively inform breeding programmes and management decisions that could enhance productivity and sustainability in heavy-weight pig production.

### Animal welfare considerations in heavy-weight pig production

While heavy-weight pig production can lead to high levels of feed consumption, inefficient feed utilisation, and elevated emissions, potential impacts on animal welfare need to be considered and addressed. A key issue that requires attention is the limited understanding of how increasing market pig weights affects pig welfare throughout the production chain, including aspects such as housing, ventilation, space allowance, handling, and transportation [53–55].

Studies have emphasised that heavy-weight pigs require increased floor space, with recommended allowances ranging from 0.89 (138 kg) to 1.3 m^2^/pig (160 kg) to optimise welfare and growth performance [56, 57]. However, optimal space allowance thresholds vary; some studies report limited effects beyond certain allowances or inconsistencies due to group size or flooring type, which limits the practical guidelines for welfare and performance [58, 59]. Methodology differences in group size, pen design, welfare measurement, and the inclusion of environmental enrichments affect the relevance of space allowance. Therefore, future research should conduct controlled, large-scale studies that isolate the effects of space allowance from group size and enrichment, focusing on heavy-weight market pigs (i.e., > 140 kg) to establish evidence-based minimum space standards.

Because of their larger size, heavy pigs easily get stuck in narrow spaces, bends and corners during handling [53]. Bertol et al. [60] and Rocha et al. [61] showed that heavier pigs (131 to 137 kg) are more difficult to handle and require more handler interventions to move forward through the alley and loading ramp compared to lighter pigs (114 to 124 kg). A similar result was observed when heavy-weight pigs (152 to 154 kg) and light-weight pigs (134 to 136 kg) were compared for handler interventions and pig behaviours [62]. Their lower ease of handling has been associated with increased salivary cortisol concentration and heart rate [63, 64]. To ease the handling of heavy-weight market pigs (150 kg) at loading, Goumon and Faucitano [65] recommended reducing the maximum ramp slope from 20°, which is usually suitable for 100 kg pigs [66], to a 15° angle. Recently, Zoratti et al. [64] evaluated the behavioural and physiological response of pigs of 122 and 153 kg negotiating ramps of different slopes (0° to 25°) and length (1.66 to 2.71 m) in a simulated loading and unloading procedure. The results showed that sloped ramps produced more slips and falls and greater blood lactate concentrations in pigs, regardless of their weight. However, results also confirmed the lower ease of handling and greater physical effort in heavier pigs, as shown by their greater reluctance to move and elevated heart rate increments compared to lighter pigs. A more recent study demonstrated that reducing the group size of pigs from 6 to 3 pigs/group, regardless of their body weight, can ease handling and improve their physiological condition [62].

Other studies [67, 68] also showed that, when mixed with unfamiliar pigs in the lairage pen, heavier pigs (140 to 145 kg) fight more than lighter pigs (70 to 120 kg). The greater fighting rate results in increased skin damage scores (mostly biting-type lesions) and greater fatigue condition at slaughter, as shown by the higher exsanguination blood creatine kinase concentrations and greater ultimate pH values in ham muscles [67, 68].

A U.S. transport survey of ~ 27,000 loads of market pigs showed a linear relationship between total transport losses (dead-on-arrival and non-ambulatory pigs) and market weight (105 to 145 kg) [69]. These animal losses can be explained by the particular vulnerability of heavier pigs to heat stress resulting from overcrowding or poorly ventilated environments [70], as they produce more body heat (+ 2% per 5 kg body weight increase) and are less able to dissipate their body heat compared with lighter pigs [71].

During transport, space allowance is one factor in particular that can affect pig welfare and stress levels, resulting in negative effects on pig survival and fitness, behaviour, physiology and meat quality [72, 73]. Space allowance requirements and recommendations can vary between countries, climatic conditions, and the weight of the animal [74]. For example, the Canadian Agri-Food Research Council recommends a minimum space allowance of 0.44 m^2^ for an average-weight slaughter pig (~ 130 kg) to ensure that it can stand and lie down to rest in their natural position without being disturbed and pushed to continually change their position during transport [75]. However, it has been demonstrated that pigs of modern genetics, being larger and heavier, may need a greater minimal space (~ 0.49 m^2^/pig of 110 kg) to entirely lie down in a full lateral recumbent position on the truck floor [76]. This new information implies that transporting pigs at the minimum space allowance presently recommended in Europe (0.425 m^2^/pig) [77] and Canada may result in overcrowding and a greater risk of bruises, injuries, fatigue, and, eventually, dead-on-arrival in heavier market-weight pigs [53, 78]. Due to their increased size, heat output, poor dissipation rate, large muscle mass, and lower cardiac output [70, 71], heavier pigs have different space requirements per unit of weight to meet their physical and thermal needs. For these reasons, the European Food Safety Authority (EFSA) Scientific Experts Committee [79] recalculated (based on allometric equation and *k*-values of 0.027 and 0.047) the minimum floor space requirements for pigs of different weights to lie down in semi- and full recumbent position. These recommendations are now being considered by the EU legislators for the amendment of the EU regulation for space allowance in area (m^2^) per pig [74, 80].

However, existing legislation or codes of practice regulating space allowance for pigs of different market weights during transport need to be science-based. To this end, Gonçalves Vero et al. [68] transported pigs of 120 and 140 kg at space allowances following the recommendations provided by the Canadian transport codes [75], i.e., 0.54 and 0.62 m^2^/pig (calculated by adding 25% extra space recommended under warm and humid conditions) in summer (17 °C on average) and 0.44 and 0.50 m^2^/pig in winter (0 °C on average), and evaluated the effects on post-transport behaviour and physiology, skin lesion scores and meat quality. No effect of the interaction between space allowance and pigs’ market weight was found in both seasons. However, these results were confounded by the short transport time (1 to 2 h) conditions, as pigs are more likely to stay standing during the initial 2 to 3 h travel [81] and cannot take advantage of the floor space they are provided with during this period.

In addition to space allowance, transport, and handling, heavier market pigs may face increased risks of lameness and limb injuries, altered disease susceptibility, and greater sensitivity to environmental conditions (e.g., ventilation capacity, temperature, humidity, and noxious gases), all of which should be considered in comprehensive welfare assessments. Future assessments of these aspects of animal welfare should be conducted and their relevance to commercial production is especially noteworthy.

### Interactive effects of immunocastration on increasing market pig weights

To reduce boar taint and aggressive behaviour, intact male pigs are commonly castrated early in life [82], but this practice raises concerns. Physical castration eliminates testosterone production, which negatively affects growth rates and feed efficiency compared to intact males [83]. However, the procedure is criticised for animal welfare reasons [84], prompting interest in alternatives to traditional castration. One such option is immunological castration, which uses a gonadotropin-releasing factor-based vaccine (e.g., Improvest^®^) to suppress testicular function late in the finishing phase. This practice is also used to manage market gilts, particularly those in heavy-weight production systems [85], by suppressing ovarian function and oestrus between the 3rd and 10th week after the second dose [86, 87]. Generally, this approach improves growth performance, carcass quality, and welfare, and is approved for commercial use in several countries [88].

The steady rise in global market pig weights, coupled with immunological castration growth worldwide (+ 8%) and in specific regions (e.g., 5%, 15%, and 30% in Germany, Belgium, and Canada, respectively) [55], requires studying the impact of immunological castration on heavy-weight pigs. Previous comprehensive meta-analyses have evaluated the effects of immunological castration on commercial male pigs [89] and Protected Designation of Origin male pigs destined for dry-cured hams [90]. Poulsen Nautrup et al. [90] confirmed that immunologically castrated males significantly gained an extra 32.54 g/d on average and require 0.234 kg less feed per kg of weight compared to physically castrated males. This meta-analysis indicates that immunologically castrated males generally consume more feed and grow faster over the entire grow-finisher period, resulting in heavier HCW compared with traditionally castrated barrows, while maintaining comparable meat quality traits. Research on immunological castration in gilts indicates no negative effects on various carcass characteristics [86, 87, 91, 92]. However, studies examining its effect on increasing market pig weights are unknown. Therefore, research into the impact of immunological castration on both sexes reared to heavy-weight endpoints is warranted.

In a study on heavy-weight immunologically castrated male pigs, Pesenti Rossi et al. [93] found that properly timed immunological castration improved welfare by reducing aggression, enhancing handling, and improving meat quality through reduced boar taint. However, immunologically castrated heavy-weight pigs approach physiological maturity while still retaining characteristics of entire males, making the timing of the effective vaccination critically important. Improperly timed vaccination can pose challenges not only for aggression and other behavioural traits but also for sexual activity, particularly in mixed-gender pens. During the 2 to 3 week pre-effective period following the second dose, pigs remain sexually active, which can temporarily increase aggressive behaviours due to the immunological response. Therefore, identifying an optimal immunological castration protocol tailored for heavy-weight pigs is essential to ensure consistent welfare and growth performance throughout the production cycle.

Regarding the effect of immunological castration on heavy-weight pigs, Comin et al. [94] found no significant differences in carcass characteristics (HCW, BFT, muscle depth, and lean meat yield) and proximate composition (moisture, protein, fat, and ash) between immunologically and traditionally castrated heavy-weight pigs with average live weights of 176 and 170 kg, respectively. However, variations in the fatty acid profiles of intramuscular fat (IMF) and subcutaneous fat between the two groups led to the conclusion that immunological castration may result in unfavourable lipid changes in fresh loin meat intended for Italian heavy pigs [94]. Harsh et al. [89] observed that variation in HCW (< 91 to > 98 kg) in immunologically castrated male pigs did not affect meat instrumental colour, firmness, drip loss, cooking loss, and Warner–Bratzler shear force (WBSF). However, they noted that as HCW increased, there was a rise in pH, subjective colour and marbling scores, and IMF content in the loins from these pigs. In contrast, Zhdanov et al. [95] reported that when pigs were reared to 136 kg body weight, there were no significant differences in ultimate pH, subjective colour and marbling scores, or water-holding capacity (WHC) between traditionally castrated males, immunologically castrated males and females, and uncastrated females.

### Carcass composition and yield in heavy-weight pigs

The impact of heavy-weight pigs on carcass characteristics, such as dressing percentage, BFT, loin eye area, muscle depth, and lean meat yield, varies considerably (Tables 2 and 3), reflecting differences in pig genetics, feeding regimes, environmental conditions, and measurement techniques.

**Table 2:** Summary of research studies demonstrating the impact of heavy-weight pigs on carcass characteristics

Trait range (average)					References
MW, kg	HCW, kg	BF depth, mm	Change (±)	Lean, %	
-	104–130	14–17	+ 3	55–53	[10]
133–172	104–138	21–28	+ 7	53–51	[11]
100–130	76–96	27–29	+ 2	-	[23]
114–122	90–97	23–24	+ 1	-	[24]
140–145	111–116	23–25	+ 2	55–54	[25]
154–215	126–178	36–50	+ 14	-	[26]
169–193	138–160	39–46	+ 7	50–47	[27]
122–136	95–107	22–23	+ 1	-	[28]
116–133	90–105	22–27	+ 5	-	[29]
120–140	93–113	25–29	+ 4	-	[30]
170–176	146–151	30–33	−3	51–52	[94]

*MW* Market weight, *HCW* Hot carcass weight, *BF* Backfat

**Table 3:** Summary of research studies demonstrating the impact of heavy-weight pigs on pork primal weight and yield

References	Class	MW, kg	Ham	Loin	Shoulder	Belly	Country
Average primal weight, kg							
[11]	Lean	133–172	12–15	14–19	11–14	8–10	USA
[24]	Non-lean	114–122	24–26	6	14–15	-	Spain
[29]	Non-lean	116–133	24–27	-	14–16	-	Spain
[30]	Non-lean	120–140	24–29	6–7	13–15	-	Spain
[31]	Non-lean	144–182	14–18	31–38	18–21	-	Italy
[96]	Lean	107–125	10–11	9–10	8–10	4–5	Canada
Average primal yield, %							
[11]	Lean	133–172	23–24	29–30	21	15–16	USA
[24]	Non-lean	114–122	26	7	15	-	Spain
[29]	Non-lean	116–133	26–27		15	-	Spain
[30]	Non-lean	120–140	25–26	6	14	-	Spain
[31]	Non-lean	144–182	24	25–26	14–15	-	Italy
[96]	Lean	107–125	25–26	23	21	11	Canada

*MW* Market weight

A simple linear regression analysis of data from 19 studies indicated that a mean increase in market weight by 10 kg can increase dressing percentage by 0.41% units [2]. Previous studies have reported increases in carcass dressing percentage of 0.20% and 1.28% units for every 10 kg increase in body weight, though these estimates come from distinct pig populations: Italian Protected Designation of Origin heavy pigs (154 to 215 kg) and conventional market-weight pigs in South Korea (115 to 135 kg), respectively [26, 97]. More recently, Metz et al. [11] found that a 10 kg increase in HCW was associated to 0.56% units increase in dressing percentage when comparing heavier carcasses (134 to 144 kg) with lighter ones (99 to 109 kg). These results disagree with Piao et al. [23], who reported no change in dressing percentage for every 10 kg increase in market weight. The discrepancy in the results between studies likely reflects differences in carcass composition and growth patterns. In most pig populations, increasing body weight is associated with greater fat deposition, particularly BFT, which contributes positively to dressing percentage due to higher carcass yield relative to non-carcass components. In contrast, Piao et al. [23] reported no significant increase in BFT with increasing body weight, suggesting a different growth trajectory in which additional weight gain may have been associated with a higher proportion of non-carcass components (such as viscera, gastrointestinal fill, or organ mass) rather than fat accretion. Furthermore, differences in genotype, feeding strategy, and production system may explain the atypical response observed by Piao et al [23]. Lean-genotype pigs or those fed protein-rich or restricted-energy diets may prioritise lean tissue deposition over fat accumulation, thereby limiting increases in dressing percentage as body weight increases. In addition, variation in gut fill at slaughter, slaughter procedures, and carcass processing methods can influence dressing percentage independently of body composition. However, it should be noted that all carcass presentations (i.e., with or without head) were consistent within each study, minimising the likelihood that presentation differences contributed to within-study variation in dressing percentage. Therefore, the negative relationship reported by Piao et al. [23] is likely context-specific and reflects differences in fat deposition patterns and non-carcass component proportions rather than a general biological trend. These findings highlight that the effect of increasing market weight on dressing percentage is not uniform and depends on the interaction between growth biology, carcass composition (particularly fat deposition), and production conditions.

It is widely recognised that BFT increases with greater weight (107 to 201 kg) as reported by numerous studies [8, 11, 20, 97–99]. A review of 25 studies reported that the average increase in BFT is 1.8 mm for every 10 kg increase in weight [2]. However, Auqui et al. [100] failed to find a significant difference in BFT (as measured at the first rib and on the *gluteus medius* muscle) between Spanish native pigs (Chato Murciano) of 147 and 176 kg on average. Differences in the anatomical location used for BFT measurements may also partially explain discrepancies among studies; however, measurement sites were consistent within each study, minimising the potential for within-study bias. This difference between studies could be largely due to the comparison between very heavy groups, where the fat deposition may have plateaued, rather than light vs. heavy groups. At heavier weights, pigs would have already reached their physiological limit for fat accretion, and differences may not be detected at similar heavier weights. Additionally, the genetic makeup of these breeds compared to commercial breeds could be a meaningful confounding factor.

A linear increase in loin eye area and muscle depth as the weight increases has also been reported [2, 11, 101]. In general, pigs with greater muscle depth tend to have larger loin eye area, and both traits can be used to estimate total carcass lean meat yield. In their review of 16 studies, Wu et al. [2] reported an increase in the loin eye area ranging from 0.1 to 2.7 cm^2^, with an average increment of 1.9 cm^2^ for every 10 kg increase in weight. Beattie et al. [102] and Piao et al. [23] reported increases of 2.4 cm^2^ and 2.2 cm^2^ in the loin eye area, respectively, for every 10 kg increase in weight. Furthermore, Metz et al. [11] reported that very heavy-weight carcasses (weighing 134 to 144 kg) had a loin eye area 10.8 cm^2^ larger than average-weight carcasses (weighing 99 to 109 kg). These effects have been attributed to the larger body size of heavy-weight pigs [2]. In another study, pigs raised to an average weight of 165 kg were found to have a significant increase in muscle depth of about 4.73 mm compared to an average HCW of 122 and 130 kg [10]. However, the authors indicated that muscle depth was significantly unaffected when the average HCW increased from 104 to 122 kg. This discrepancy may result from inadequate protein and amino acid levels in the diet for pigs with live weights exceeding 130 kg, which demonstrates the interactive effect of weight and diet described above.

Heavy-weight pigs have been reported to produce larger primal and cut weights, albeit with a decrease in some primal yields when expressed as a percentage of carcass weight (Table 3). Latorre et al. [30] showed that the weight of total trimmed primal cuts increases linearly by 3.63 kg for every 10 kg increase in weight above 120 kg in pigs intended for dry-cured ham production. In addition, trimmed ham and loin yields decrease as weight rises from 120 to 140 kg, while the trimmed shoulder yield remains unaffected as weight increases. Consequently, despite increases in total primal cut weight, higher carcass weights may negatively affect the industrial appeal of cuts other than ham due to increased fat deposition. For bellies, a meta-analysis of data by Wu et al. [2] found that belly yield increases by 0.09% to 0.61% points for every 10 kg increase in weight. Table 3 summarises the effects of weight on primal cut weights and yields based on various studies involving lean-type and fat-type pigs from different countries. It is apparent that lean-genotype pigs, commonly referred to as market weight pigs, regardless of the weight, such as those in the studies by Correa et al. [96] and Metz et al. [11], generally have lower market and primal cut weights compared to fat-type genotype pigs. Fat-type genotype pigs, such as those studied by Serrano et al. [103], exhibit heavier primal cuts, particularly the ham. The average primal yield percentages further indicate that lean-genotype pigs tend to have higher yields for the loin and shoulder cuts, while fat-type genotype pigs produce higher yields for ham. The increased yield values for ham in fat-type genotype pigs can largely be attributed to selection for their primary purpose of producing dry-cured hams. It is also worth noting that differences in primal yield between lean-type and fat-type genotype pigs reflect their maturation patterns, with late-maturing lean pigs favouring loin and shoulder development, while early-maturing fat-type pigs yield more ham due to earlier fat deposition. In this context, the distinction between lean- and fat-type pigs refers to genetic background rather than age or slaughter weight, with lean-type pigs representing genetically improved commercial lines and fat-type pigs typically corresponding to local or traditional breeds that have undergone limited selection for lean growth.

Generally, total carcass lean meat yield decreases with increasing weight [10, 11, 27] due to increased overall fat content [2]. In a few cases, such as the study by Piao et al. [23], no significant change is reported in lean meat yield when weights increased from 100 to 130 kg. The observed difference may be attributed to genetic differences, feeding strategies, or growth patterns that promote lean tissue accretion even at heavier weights. However, in general, it has been established that as pigs attain heavier weights > 130 kg, there is a reduction in lean meat yield with increasing weight [2].

### Chilling dynamics in heavy commercial carcasses

In commercial practice, pig carcasses are chilled using one of several independent systems, most commonly conventional, blast, or spray chilling. The conventional chilling systems operate at refrigerated temperatures (i.e., 1 °C to 4 °C) with moderate air velocities (0.1 to 0.8 m/s), requiring 18 to 24 h to complete the chilling process. Blast chilling systems first pre-chill carcasses for ~ 90 min at temperatures between −20 °C and −35 °C with high air velocities of 3 to 5 m/s, followed by spray chilling at temperatures between 1 °C and 4 °C [104]. These systems were originally designed for light-weight carcasses, but the industry trend toward heavy-weight pigs has introduced new challenges in chilling dynamics and management of meat quality. As carcass size increases, the surface area-to-volume ratio decreases, resulting in reduced cooling efficiency and slower temperature decline [105–107]. This explains the contribution of HCW on carcass chilling rate, accounting for 25% of its variation during the first 5 h and 32% between 5 and 13 h postmortem in a blast chilling system [10].

The number of studies evaluating the impact of heavy carcass weights on chilling rate is limited. Overholt et al. [107] reported delays of ~ 17 and ~ 27 h to chill loins to 2 °C in 95 and 105 kg carcasses, respectively, in a blast chilling system. Chilling rate for hams showed the same delay pattern, with internal temperatures of 2 °C being reached in 21 h in 95 kg carcasses and in 28 h in 105 kg carcasses. In a more recent study, lighter carcasses (< 112 kg) were 3.5 °C colder than heavier carcasses (> 124 kg) at 5 h postmortem chilling, and after 17 h postmortem, lighter carcasses were still colder (−0.73 °C) compared to heavier ones [10]. In the context of actual differences in postmortem temperature, Price et al. [10] categorised carcasses into slow-, medium-, and fast-chilling groups irrespective of HCW, with those in the slow category being 13.7 kg heavier than the fast-chilling carcasses. The authors reported that at 45 min postmortem, the slow-chilling carcasses were 0.86 °C warmer than the fast-chilling carcasses. Furthermore, at 5 and 17 h postmortem, fast-chilling carcass temperatures were 4.85 and 1.48 °C lower, respectively, than the slow-chilling ones. Comparing different primal cuts, the loin cools the fastest, followed by the shoulder, while the ham shows the slowest chilling rate among the primal cuts between 3 and 21 h postmortem under conventional chilling [108].

The interactive effect of heavier HCW and chilling rates on pork meat quality remains underexplored. Price et al. [10] demonstrated that heavier carcasses (125 kg) subjected to slower chilling rates yielded more tender loin chops, with reduced slice shear force values (−0.82 and −2.04 kg) across endpoint temperatures of 63 °C and 71 °C, respectively, compared to lighter carcasses (111 kg). These findings suggest that while heavier carcasses may require longer chilling times, the resulting improvements in meat tenderness could enhance product value, highlighting the need to reconsider chilling strategies in response to evolving carcass characteristics. Based on the relationship between chilling rate, pH decline, and WHC, slower chilling rates observed in heavier carcasses result in a potential reduction in WHC [10], with slow-chilling carcasses presenting 1.14% and 0.64% reduction in cooking loss and purge loss, respectively, compared to fast-chilling carcasses.

Carcass composition also influences the chilling rate. Greater BFT may reduce shrinkage or chilling losses in heavier carcasses, likely due to a smaller surface area and/or greater subcutaneous fat coverage [105, 109].

### Relationship between increasing market pig weight and muscle fibre

Generally, pig skeletal muscle fibres are grouped under type I slow-twitch oxidative, type IIA fast-twitch oxido-glycolytic, or type IIB fast-twitch glycolytic, based on their contractile and metabolic properties [110]. It is well established that the number of muscle fibres remains unchanged postnatally, whereas muscle volume increases due to the enlargement of existing fibres. Specifically, from birth until eight weeks postnatal, the proportion of type I fibres in the *flexor digitorum superficialis*, *vastus intermedius*, and *soleus* muscles of pigs continues to increase [111]. In contrast, the authors noted that fast-twitch fibres in the hind limb muscles differentiate into type IIA and IIB fibres.

The heterogeneity of muscle fibre characteristics (number, size, and composition) varies depending, among others, on the market pig weight [112–115].

Market pig weight and muscle fibre composition interact primarily through fibre hypertrophy and muscle-specific shifts in fibre proportions, producing complex and sometimes muscle-dependent impacts on final pork quality. Previous studies have shown that increasing weight to 130 kg significantly increases the cross-sectional area of all fibre types [112, 113, 116], with muscle glycolytic potential remaining unaffected, though [117]. The latter evidence does not support the theory of a greater glycolytic potential in heavy-weight pigs (> 170 kg), as formulated by Nanni Costa et al. [118], to explain their lower risk of producing dark, firm, and dry pork after overnight lairage. Collectively, these findings indicate that skeletal muscle in pigs increases in size during growth, with a relatively constant rate of enlargement between 25 and 90 kg, followed by a reduced growth rate beyond 90 kg [119]. Čandek-Potokar et al. [112] reported that, as weight increased from 100 to 130 kg, the cross-sectional area of type I and type IIB fibres significantly increased, while the numerical and area proportions of all fibre types remained unchanged. Similarly, Choi and Oh [116] observed a significant increase in the cross-sectional area of type IIA and type IIB fibres as weight increased from 96 to 130 kg, with no corresponding change in the numerical or area percentages of any fibre type. Even at heavier HCW (97 to 133 kg), no significant differences were found in the percentages or total areas of fibre types in the *longissimus lumborum* muscle, except for type IIA, whose total fibre area tended to decrease as HCW increased [8]. Taken together, these findings are consistent with Jeong et al. [113], who reported that muscle fibres primarily grow through hypertrophy during the late finishing phase, while other fibre characteristics, aside from size, are not significantly affected by body weight.

### Effects of weight on pork lean and fat quality, and sensory characteristics

The effect of increasing weight on pork quality and sensory attributes has been widely studied, with findings showing both consistent patterns and some discrepancies (Table 4). The effects of increasing weight (115 to 150 kg) on ultimate pH as assessed in the *longissimus* muscle range from none [8, 97] to a decline [2, 7] or a slight (≥ 0.10 units) increase [101, 120]. Similarly, the effects on the ultimate pH in ham muscles, such as the *gluteus medius*, *semimembranosus*, and *biceps femoris* muscles, range from none [8, 97] to a 0.20-unit increase [120]. The discrepancies across studies could be ascribed to genotypic differences in growth patterns, variation in muscle fibre type and metabolism, and different pre-slaughter conditions triggering a different physiological stress response, all influencing muscle fibre glycogen reserves, and consequently postmortem pH decline.

**Table 4:** Summary of research studies demonstrating the impact of increasing market weight (MW) on pork quality

References	MW, kg	HCW, kg	pH (24 h)	L*	a*	b*	DL/PL, %	Marbling	IMF, %	CL, %	SF, kg
[7]		53–130	↓	↓	↑		NS	NS		↓	↓
[8]		78–145	NS	NS	NS	↑		NS		↓	↓
[10]		104, 116, 122, 130	NS	NS			↓			↓	
[97]	115, 125, 135		NS	NS	NS		NS		NS	NS	NS
[99]		≤ 90, 91–100, > 100	NS	NS	↑	NS	NS			NS	↑
[100]	148, 176	118, 141	NS	NS	NS	↑			↑		
[101]	111.6, 150.1		↑	NS	NS	NS	NS		↑	↓	↓
[120]	110, 130		↑						NS		
[121]		< 111.8, 111.8–119.1, 119.2–124.4, > 124.4	NS	NS	NS	NS	NS	NS	NS	NS	NS
[122]	100, 110, 120		NS	NS	↑	↑				↓	NS

*MW* Market weight, *HCW* Hot carcass weight, *DL/PL* Drip/purge loss, *IMF* Intramuscular fat, *CL* Cooking loss, *SF* Shear force, *NS* Non-significant, ↑ Increase, ↓ Decrease

The effects of increased weight on pork colour, which is an important factor influencing the consumers' purchase intent [123, 124], and which varies according to multiple interacting factors, including muscle fibre type and metabolic properties, pre-slaughter stress, chilling rate, postmortem pH decline, intramuscular fat content, and processing conditions, all of which may contribute to the variability observed across studies. Specifically, colour is closely related with the ultimate pH value, with higher pH being associated with darker meat (lower Minolta L* values) [123, 125]. In agreement with previously reported inconsistencies in the results [2], the results related to instrumental colour parameters (L*, a*, b*) and subjective colour scores as assessed in the *longissimus* muscle from most recent studies range from no effect [8, 10, 97, 99, 100, 121] to a minor reduction in the L* value (−0.24 units) and increase in the a* and b* values (+ 0.11 and 0.09 units, respectively) for every 10 kg increase in HCW [7, 8]. A similar variation has also been reported in the ham muscles, with effects ranging from none [8, 26] to a slight reduction in the a* and b* values in cured ham steaks from heavier pigs [12].

Although earlier reports suggested inconsistencies [2], more recent studies generally agree that increasing weight (up to 150 kg) does not affect drip or purge losses [7, 97, 99, 101, 121]. However, Price et al. [10] noted a 0.62% reduction in purge loss with increasing HCW (up to > 124.4 kg).

Several studies have demonstrated reduced cooking losses with heavier weights [7, 8, 10, 101]. The reported effects range from approximately −0.5% (after 63 °C to 71 °C cooking temperature) per 10 kg increase in HCW [7, 8] to −7.3% cooking loss reduction in loin chops of 150 kg pigs compared to 112 kg pigs [101]. However, other studies [97, 99, 121] reported no significant differences in cooking loss values across weight groups. The mixed findings may be due to variations in weight ranges, meat ageing duration, endpoint cooking temperatures, and genotype. Most studies that use larger weight differences and longer postmortem ageing time (i.e., 14 and 21 d) more often detect reductions, while those with narrower ranges or shorter ageing durations (i.e., 2 d) do not.

Recent findings generally show that tenderness improves or remains stable with increasing weight. Harsh et al. [7] and Price et al. [8] recorded decreases of 1.27 and 0.67 kg, respectively, in pork slice shear force values for each 10 kg increase in HCW. Li et al. [101] found approximately a 43% reduction in WBSF values when comparing pigs marketed at 150 kg vs. 112 kg. Similarly, Price et al. [10] reported that pork shear force value decreased by 2.0 kg as HCW increased (up to > 124.4 kg). However, other studies [97, 117, 121, 122] reported no changes in WBSF values with increasing liveweight (≥ 125 kg). The discrepancy in the results between studies may be explained by the difference in the weight ranges or heavier endpoints, where meat compositional shifts, such as increased IMF and altered connective tissue solubility, are more pronounced, making a reduction in instrumental tenderness detectable, and cooking endpoint temperature, with effect of weight on pork shear force being observed when loin chops were cooked to 71 °C compared to 63 °C [10].

Marbling score and IMF are important traits influencing sensory evaluation and consumer acceptance [126]. Generally, IMF increases with a higher weight [2, 100, 101]. For instance, Auqui et al. [100] found a 1.38% IMF increase in heavier pigs from 148 to 176 kg, and Li et al. [101] reported a 0.43% rise from 112 to 150 kg. Nonetheless, a number of other studies [96, 97, 120, 121] found no significant changes in IMF. The reason for these discrepancies may be genetic differences, gender, maturity stage, and nutrient partitioning, as some genotypes or late-maturing pigs continue to deposit lean tissue at higher weights rather than shifting towards fat deposition. Additionally, visual marbling scores of loin and ham muscles generally remained unchanged across weight groups [7, 8, 26, 96, 121], although greater marbling scores have been reported in the ventral loin surface of heavier carcasses weighing 130 kg [7].

Studies on the impact of increased weight on fat quality and composition have been mainly focused on iodine value, fatty acid composition, and fat firmness [127]. Harsh et al. [7] and Price et al. [8] reported reductions of the iodine value by 1.26 and 0.92 units, respectively, in the shoulder blade backfat per 10 kg increase, with HCW (53 to 145 kg) explaining 7% to 10% of iodine value variation. Another study showed a reduction of the belly adipose tissue iodine value by 2.26 to 2.45 units, resulting in firmer bellies, as HCW increased from 99–109 to 134–144 kg [12]. However, previously Correa et al. [98] failed to find an effect of weight on the iodine value in the belly of pigs of a lower weight range (107 to 125 kg). Discrepancies may relate to weight range and anatomical location. Although the reported iodine values remained below the industry threshold value of 74, an indicator of softer fat, in all studies [127], in general, the results, regardless of the anatomical location, show that fat firmness increases with increasing weight.

Ba et al. [122] reported saturated fatty acid (SFA) reduction by 2.99% unit in the *longissimus dorsi* muscle per 10 kg weight gain (100 to 120 kg), while Oh et al. [97] observed a 2.25% unit reduction in SFA percentage in the *semimembranosus* muscle with weight increase from 115 to 135 kg. The differences in weight range sensitivity, breed, or diet may explain the discrepancy. No significant changes in monounsaturated fatty acids (MUFA) were observed in adipose tissues from the ham, belly, loin or IMF with increasing weight [2, 12, 100, 122]. Same as Correa et al. [98] in bellies of pigs up to 125 kg, Metz et al. [12] found a tendency (*P* = 0.06) for decreased polyunsaturated fatty acids (PUFA) in the belly fat as HCW increased from 99 to 144 kg. Conversely, Ba et al. [122] reported a 3.98% unit increase in PUFA in the *longissimus dorsi* muscle IMF for every 10 kg weight gain (100 to 120 kg), agreeing with Auqui et al. [100], who also found higher PUFA levels in the *longissimus dorsi* muscle IMF of 176 kg pigs compared to 148 kg pigs. Differences may stem from fat tissue location (belly vs. IMF), lipid metabolism enzyme activity variations [128], and dietary PUFA intake [100, 122] versus limited de novo PUFA synthesis [12, 98] and pigs’ genetic background. For example, in Chato Murciano pigs, a Spanish local breed, the *longissimus dorsi* muscle IMF showed lower SFA and higher PUFA levels at 176 kg compared to 148 kg [100].

The impact of weight on pork meat sensory attributes is unclear [2]. Unlike Huff-Lonergan et al. [125], who failed to find a correlation between HCW and tenderness or flavour, Hwang et al. [99] reported a significant correlation between HCW (≤ 90 to > 100 kg) and these sensory traits, with tenderness negatively correlated (*r*_*p*_ −0.70; *P* < 0.001) and flavour positively correlated (*r*_*p*_ 0.59; *P* < 0.001). Sensory tenderness scores of 2-d [121] and 14-d [10] aged chops, cooked to different endpoint temperatures (63 and 71 °C), improved with an increase in HCW (< 112 to > 124 kg), whereas Hwang et al. [99] noted a decrease with HCW. Some studies observed no significant change [97, 122] when slaughter weight increased from 100 to 135 kg. Duan et al. [117] reported a greater pork toughness when slow-growing gilts were raised to 125 kg, while no such difference was found in the 107 and 115 kg classes. The conflicting findings can be attributed to several interacting factors, such as postmortem ageing time and enzyme activities, endpoint cooking temperature, chilling rate, muscle type, fibre composition, genetic background, and feeding regimes. Notwithstanding these inconsistencies, the prevalent trend in the literature, particularly in studies involving larger weight ranges, indicates that sensory tenderness will likely improve with increasing weight.

Wu et al. [2] reported mixed findings regarding the effects of increasing weight on pork juiciness. More recent studies confirm these conflicting results, with juiciness scores ranging from increased with weight to unaffected. In Duan et al. [117] study, panellists detected an improvement in pork juiciness when pig body weight was increased from 107 to 115 kg, but this difference disappeared at 125 kg body weight. Ba et al. [122] reported an increase in juiciness when the weight rose from 100 to 120 kg. More specifically, Rice et al. [121] found increased juiciness scores (+ 4.6 units) in loin chops from medium-heavy HCW group (119 to 124 kg) compared to chops from the lightweight HCW group (< 112 kg). More recently, Price et al. [10] reported a 0.24-unit increase in loin chops' juiciness score for every 10 kg increase from < 112 to > 124 kg HCW. Conversely, a number of other studies failed to find an effect of weight on pork juiciness [10, 97, 99]. The incongruence in the results between studies can be explained by the applied meat cooking endpoint temperature [129], with effects being found when meat chops were cooked at an endpoint temperature of 71 °C [10, 121] compared to 63 °C [10, 97, 99], regardless of the weight category.

An increase in market pig weight has been associated with enhanced pork flavour. One possible cause is elevated levels of unsaturated fatty acid oxidative aldehydes [122], with increased flavour scores being reported in pork from heavier weight (up to 125 kg) [99, 117, 122]. These results disagree with a number of other studies that did not find any effect of weight on this sensory trait [10, 97, 121]. The magnitude of difference in weight between studies may partly explain the discrepancies.

There is evidence about a positive relationship between pork off-flavour and HCW [125] that can be attributed to the greater fat deposition as HCW increases, along with higher PUFA concentration in the fat [2, 98], and the likelihood of the presence of off-odour compounds, particularly skatole, in the fat of castrated male pigs and gilts. However, Rice et al. [121] did not find any effect of increased weight on pork off-flavours, which may be due to the lack of differences in visual marbling score and IMF concentration.

### Genetic selection for increased performance

In the United States and Canada, genetic progress has been driven by long-term selection for increased growth rate, feed efficiency, and lean gain, supported more recently by genomic selection tools, resulting in modern commercial pig lines capable of sustaining efficient lean deposition to heavier market weights while maintaining acceptable carcass composition and production efficiency.

Over recent decades, numerous studies have documented the influence of genetics on performance, carcass and meat quality traits in pigs [130–133]. Regarding growth performance traits in swine, studies have reported variable heritabilities for ADG (*h*^*2*^ 0.13 to 0.55), ADFI (*h*^*2*^ 0.24 to 0.66), and feed conversion ratio (*h*^*2*^ 0.17 to 0.39), primarily due to breed variation and trait definition (Table 5) [131, 134–136]. Moderate to high heritabilities have also been reported for carcass merit traits, such as BFT (*h*^*2*^ 0.31 to 0.66) and muscle depth (*h*^*2*^ 0.15 to 0.63) [130, 131, 134, 137–142], and relevant meat quality attributes, such as IMF (*h*^*2*^ 0.23 to 0.54) and drip loss (*h*^*2*^ 0.21 to 0.28) [130, 131, 138, 143, 144]. These recorded magnitudes of heritability estimates indicate the feasibility of genetic improvement in swine for these traits.

**Table 5:** Reported heritability estimates for growth, carcass, and meat quality traits in pigs

Trait	Heritability range (*h*^2^)	References
Growth performance		
Average daily gain	0.13–0.55	[131, 134–136]
Average daily feed intake	0.24–0.66	[134–136]
Feed conversion ratio	0.17–0.39	[131, 134–136]
Carcass traits		
Hot carcass weight	0.10–0.39	[130, 137–139]
Market weight	0.26–0.38	[131, 138]
Backfat thickness	0.31–0.66	[130, 131, 134, 136–143]
Muscle depth	0.15–0.63	[130, 131, 134, 137–139]
Loin eye area	0.22–0.62	[130, 138, 140]
Carcass length	0.51	[130]
Ham weight (intact)	0.14–0.57	[130, 137, 138, 140]
Loin weight (intact)	0.18–0.63	[130, 137, 138, 140]
Belly weight (intact)	0.16–0.51	[130, 137, 138, 140]
Shoulder weight (intact)	0.22–0.55	[130, 138]
Butt weight (intact)	0.09–0.29	[130, 137, 138, 140]
Picnic weight (intact)	0.12–0.44	[130, 137, 140]
Quality traits		
L*	0.11–0.36	[130, 137–139, 144]
a*	0.14–0.36	[130, 137, 138, 144]
b*	0.04–0.32	[130, 137, 138, 144]
Subjective colour score	0.14–0.26	[137, 138, 144]
Subjective marbling score	0.11–0.47	[130, 137–139, 144]
pH	0.06–0.39	[130, 137–139, 144]
Intramuscular fat	0.23–0.54	[131, 134, 137, 139, 143, 144]
Drip loss	0.21–0.28	[130, 138]
Cooking loss	0.20	[130]
Slice/Warner–Bratzler shear forces	0.19–0.39	[130, 137, 144]

Most breeding programs for commercial swine production focus on optimising feed efficiency, carcass and meat quality traits at a target market weight of 115 to 125 kg, rather than maximising all traits simultaneously, given the inherent trade-offs involved, particularly with IMF [130, 145–147]. However, the consequences of slaughtering pigs at weights exceeding this target on economically important traits, as well as the potential need to adjust current selection objectives accordingly, remain largely unknown. Genetic × environment interactions, including breed and maturity (early vs. late) interactions, play a role in phenotypic changes associated with weight. For instance, lean-type breeds show rapid muscle area increases that plateau earlier, while fatty-type breeds are earlier maturing with decreased muscle accretion/increased fat deposition earlier than lean, genetically improved breeds [133]. Commercial lean, fast-growing breeds, such as Landrace and a crossbreed Large White x Yorkshire, have been favoured by the swine sector for the efficient production of commercial pigs, while crossbreeding with terminal sire lines, such as Duroc, has often been used to enhance meat quality attributes. Previous studies have shown how phenotype means change with heavier weight and which direct traits and measurements are practical for genetic selection [11, 29]. Slaughtering crossbred pigs at 20 to 40 kg above the weight used in purebred selection indices has the potential to lead to, among other effects, a decrease in growth and feed efficiency, as well as an increase in BFT and a decrease in lean meat yield.

The relative weight of individual pork primal and subprimal cuts is also influenced by genetics, with reported variability in heritability estimates (*h*^*2*^ 0.09 to 0.63; Table 5) [130, 137, 138, 140]. On the other hand, the genetic correlations among these traits are moderate to high (*r*_*g*_ −0.17 to 0.83) [130, 137, 138, 140]. Additionally, their phenotypic relationships, as well as the fat:lean ratio of each component of the pig carcass, have been reported to change depending on the stage of pig development [133, 148, 149]. Therefore, studies show that growing pigs above the weight targeted during the breeding selection process could lead to changes not only in the relative proportion of primal cuts but also in their composition and, consequently, impact their value and acceptability.

Moderate to strong correlations observed among some performance, carcass and meat quality traits also need to be considered (Table 6) [130, 136, 138], as producers’ profitability is influenced by production costs and carcass pricing. For example, meat quality traits such as IMF content exhibit antagonistic relationships with leanness (*r*_*p*_ −0.55 to −0.27) [150] but positive correlations with economically important traits at the processing plant level, such as belly fat (*r*_*g*_ 0.76) and firmness (*r*_*g*_ 0.66) [143], requiring balanced selection strategies to make the production of heavy-weight pigs profitable for the entire sector.

**Table 6:** Reported genetic correlations for growth, carcass, and meat quality traits in pigs

Traits	Genetic correlation range (*r*_*g*_)	References
Growth performance		
ADG × FCR	−0.21 to −0.59	[134–136, 141]
ADG × ADFI	0.32 to 0.63	[134, 136, 141]
Carcass traits		
Ham × Loin	0.20 to 0.77	[130, 137, 138, 140]
Ham × Belly	−0.17 to 0.71	[130, 137, 138, 140]
Loin × Belly	−0.42 to 0.83	[130, 137, 138, 140]
Shoulder × Belly	−0.61 to 0.44	[130, 137, 138, 140]
Shoulder × Ham	0.83	[130, 137, 138, 140]
Shoulder × Loin	−0.27	[130, 137, 138, 140]
Picnic × Butt	0.38 to 0.78	[130, 137, 138, 140]
HCW × BFT	0.12 to 0.39	[130, 138]
Carcass length × HCW	0.89	[130]
Carcass length × Loin eye area	0.47	[130]
Muscle depth × HCW	−0.36 to 0.77	[130, 131, 138]
Muscle depth × Market weight	−0.40 to 0.37	[130, 131, 138]
Muscle depth × BFT	−0.34 to −0.32	[130, 131, 138]
Quality traits		
Colour score × L*	−0.84 to −0.70	[137, 138, 144]
Colour score × a*	0.13 to 0.45	[137, 138, 144]
Colour score × b*	−0.93 to −0.45	[137, 138, 144]
IMF × Marbling score	0.58 to 0.77	[137, 139]
IMF × a*	0.26	[137, 139, 144]
IMF × L*	0.96	[137, 139, 144]
pH × L*	−0.90 to −0.34	[130, 137–139]
pH × a*	−0.38 to −0.27	[130, 137–139]
pH × b*	−0.64 to −0.59	[130, 137–139]

*ADG* Average daily gain, *FCR* Feed conversion ratio, *ADFI* Average daily feed intake, *HCW* Hot carcass weight, *BFT* Backfat thickness, *IMF* Intramuscular fat

Furthermore, the use of genomics has opened new opportunities for enhanced genomic selection of specific traits within a population [151, 152], enabling the combination of data from purebred and crossbred pigs. Over the years, multiple candidate genes have been identified that may play a significant role in performance, carcass, and meat quality-related traits based on their known function. For instance, a Genome-Wide Association Study (GWAS) found candidate genes linked to feed conversion ratio in Landrace (nucleosome assembly protein 1-like 4: *NAP1L4*, lymphocyte-specific protein 1: *LSP1*, and PTPRF interacting protein alpha 1: *PPFIA1*), Duroc and Large White (minichromosome maintenance complex component 6: *MCM6* and zinc finger RAN-binding domain-containing protein: *ZRANB3*) [136, 153]. Other studies have identified candidate genes with potential pleiotropic effects on ADG. For instance, PH domain leucine-rich repeat protein phosphatase-1 (*PHLPP1*) was associated with both ADG and lean meat yield in North American Duroc pigs [154], while MyoD family inhibitor domain-containing protein (*MDFIC*), ribosomal protein S12 (*RPS12*), phosphodiesterase 4D (*PDE4D*), and aquaporin-4 (*AQP4*) were putatively linked to ADG and body weight in Large White and Landrace pigs [142]. As pigs grow heavier, the expression of fat-related genes becomes more pronounced, and recent large-scale functional genomic studies have identified several novel candidate genes, such as 7-dehydrocholesterol reductase (*DHCR7*), fibroblast growth factor 23 (*FGF23*), mesenteric estrogen-dependent adipogenesis gene (*MEDAG*), diacylglycerol kinase 1 (*DGK1*), and pleiotrophin (*PTN*), that are associated with BFT across diverse purebred populations [155]. In Canadian commercial crossbred pigs, genes with pleiotropic effects, such as melanocortin-4 receptor (*MC4R*) and phorbol-12-myristate-13-acetate-induced protein 1 (*PMAIP1*), were associated with BFT, total fat, and fat in all primal cuts [146], suggesting that increasing market pig weights may amplify the effect of these genes. Another study using a four-way crossbred pig population and specific-locus amplified fragment sequencing GWAS identified phospholipase B domain-containing protein 2 (*PLBD2*) as a strong candidate gene for lean meat yield, with phosphoglucomutase 2-like 1 (*PGM2L1*) showing pleiotropic effects on lean meat yield and BFT traits [156]. Research in commercial crossbred pigs using a combined GWAS approach identified key genes (protein phosphatase 3 catalytic subunit alpha: *PPP3CA*, cytoplasmic polyadenylation element binding protein 4 (*CPEB4*), enoyl-CoA hydratase 1: *ECH1*, fibroblast growth factor receptor-like 1: *FGFRL1*, and carbohydrate sulfotransferase 11: *CHST11*) associated with primal cut weights [157]. In addition, the genes ST8 alpha-N-acetyl-neuraminide alpha-2,8-sialyltransferase 6 (*ST8SIA6*) and ankyrin-repeat and fibronectin-type III domain containing 1 (*ANKFN1*) have been associated with IMF and marbling score, respectively [158]. Some functional candidate genes identified tend to vary across breeds, highlighting breed-specific genetic architectures. However, since the end goal of breeding programs is typically the production of commercially crossbred pigs, and given the steady increase in market pig weights, further research is needed in heavy commercial crossbred populations. This would help uncover key functional candidate genes associated with heavier weights, ultimately supporting more targeted and effective breeding strategies for the swine industry.

## Conclusions, research gaps and future perspective

Raising pigs to heavier market weights influences growth efficiency, carcass composition, and meat quality, but current evidence shows that research is not yet adequate to guide nutrition, management, or genetic improvement for pigs exceeding 135 kg, particularly in North America. However, it is worth noting that substantial research, particularly from Italian heavy pig production systems, has advanced understanding of the relationships among slaughter weight, muscle biology, and meat quality. Critical knowledge gaps remain in defining nutrient requirements for heavy pigs, understanding how immunological castration affects carcass composition and quality at heavier weights, and determining how heavier carcasses with greater fat cover interact with chilling rate to influence meat quality. In addition, it remains unclear whether pigs slaughtered at heavier weights (≥ 150–170 kg) undergo muscle-specific metabolic or fibre-type transitions that may influence pork meat quality. Therefore, empirically validating the relationship between heavy-weight pigs, fibre-type metabolism, and quality outcomes will be worthwhile. Genetic selection strategies have not fully adapted to the biological and carcass characteristics of heavy-weight pigs, and selection accuracy continues to be limited by insufficient genomic and phenotypic integration for meat quality traits. Finally, the economic value of genomic selection for heavy pigs remains under-evaluated. Addressing these gaps through targeted nutritional trials, refined breeding objectives, improved genomic prediction resources, and robust economic analyses will be essential for optimising the performance and quality of pigs marketed at increasingly heavy weights.

## Data Availability

No datasets were generated or analysed during the current study.

## Abbreviations

ADFI: Average daily feed intake
ADG: Average daily gain
ANKFN1: Ankyrin-repeat and fibronectin-type III domain containing 1
AQP4: Aquaporin-4
BFT: Backfat thickness
CHST11: Carbohydrate sulfotransferase 11
CPEB4: Cytoplasmic polyadenylation element binding protein 4
DGK1: Diacylglycerol kinase 1
DHCR7: 7-Dehydrocholesterol reductase
ECH1: Enoyl-CoA hydratase 1
EFSA: European food safety authority
EU: European union
FGF23: Fibroblast growth factor 23
FGFRL1: Fibroblast growth factor receptor-like 1
G:F: Gain-to-feed ratio
GWAS: Genome-wide association studies
*h*^2^: Heritability
HCW: Hot carcass weight
IMF: Intramuscular fat
LSP1: Lymphocyte-specific protein 1
MC4R: Melanocortin-4 receptor
MCM6: Minichromosome maintenance complex component 6
MDFIC: MyoD family inhibitor domain-containing protein
MEDAG: Mesenteric estrogen-dependent adipogenesis gene
MUFA: Monounsaturated fatty acid
NAP1L4: Nucleosome assembly protein 1-like 4
NRC: National research council
PDE4D: Phosphodiesterase 4D
PGM2L1: Phosphoglucomutase 2-like 1
PHLPP1: PH domain and leucine-rich repeat protein phosphatase 1
PLBD2: Phospholipase B domain-containing protein 2
PMAIP1: Phorbol-12-myristate-13-acetate–induced protein 1
PPFIA1: PTPRF interacting protein alpha 1
PPP3CA: Protein phosphatase 3 catalytic subunit alpha
PTN: Pleiotrophin
PUFA: Polyunsaturated fatty acid
*r*_g_: Genetic correlation
*r*_p_: Phenotypic correlation
RPS12: Ribosomal protein S12
SFA: Saturated fatty acid
SID: Standardised ileal digestible
ST8SIA6: ST8 Alpha-N-acetyl-neuraminide alpha-2,8-sialyltransferase 6
WBSF: Warner–Bratzler shear force
WHC: Water-holding capacity
ZRANB3: Zinc finger ran-binding domain-containing protein 3
